# Formulation for Oral Delivery of Lactoferrin Based on Bovine Serum Albumin and Tannic Acid Multilayer Microcapsules

**DOI:** 10.1038/srep44159

**Published:** 2017-03-10

**Authors:** Ece Kilic, Marina V. Novoselova, Su Hui Lim, Nikolay A. Pyataev, Sergey I. Pinyaev, Oleg A. Kulikov, Olga A. Sindeeva, Oksana A. Mayorova, Regan Murney, Maria N. Antipina, Brendan Haigh, Gleb B. Sukhorukov, Maxim V. Kiryukhin

**Affiliations:** 1 Institute of Materials Research and Engineering, Agency for Science, Technology and Research (A*STAR), 2 Fusionopolis Way, Innovis, #08-03, 138634, Singapore; 2 Food Science and Technology Programme, Department of Chemistry, National University of Singapore, 3 Science Drive 3, 117543, Singapore; 3 N.G. Chernyshevsky Saratov State University, 83 Astrakhanskaya Street, Saratov, 410012, Russia; 4 National Research Ogarev Mordovia State University, 68 Bolshevistskaya Str., Saransk, Republic of Mordovia, 430005, Russia; 5 School of Engineering and Materials Science, Queen Mary University of London, Mile End Road, London E1 4NS, United Kingdom; 6 AgResearch Limited, Ruakura Research Centre, Bisley Road, Private Bag 3123, Hamilton 3240, New Zealand

## Abstract

Lactoferrin (Lf) has considerable potential as a functional ingredient in food, cosmetic and pharmaceutical applications. However, the bioavailability of Lf is limited as it is susceptible to digestive enzymes in gastrointestinal tract. The shells comprising alternate layers of bovine serum albumin (BSA) and tannic acid (TA) were tested as Lf encapsulation system for oral administration. Lf absorption by freshly prepared porous 3 μm CaCO_3_ particles followed by Layer-by-Layer assembly of the BSA-TA shells and dissolution of the CaCO_3_ cores was suggested as the most efficient and harmless Lf loading method. The microcapsules showed high stability in gastric conditions and effectively protected encapsulated proteins from digestion. Protective efficiency was found to be 76 ± 6% and 85 ± 2%, for (BSA-TA)_4_ and (BSA-TA)_8_ shells, respectively. The transit of Lf along the gastrointestinal tract (GIT) of mice was followed *in vivo* and *ex vivo* using NIR luminescence. We have demonstrated that microcapsules released Lf in small intestine allowing 6.5 times higher concentration than in control group dosed with the same amount of free Lf. Significant amounts of Lf released from microcapsules were then absorbed into bloodstream and accumulated in liver. Suggested encapsulation system has a great potential for functional foods providing lactoferrin.

Lactoferrin (Lf) is a whey protein that has considerable potential as a functional ingredient in food, cosmetic and pharmaceutical applications. Lf possesses various biological functions such as antibacterial, antiviral, antitumor, antifungal, anti-inflammatory, immunomodulatory, analgesic, antioxidant, enhancement of lipid metabolism^1^,^2^,^3^,^4^,^5^,^6^. In addition, it is low-cost and displays no toxic side effects. The major challenge that hinders its wide application is its poor *in vivo* stability due to rapid degradation by proteolytic enzymes. When intravenously injected, the plasma half-life of Lf is several minutes and frequent administration is necessary to achieve a therapeutic effect^2^. Oral administration is natural and non-invasive way for supplementing Lf, since important receptors of Lf in the body are the intestinal mucosa and gut-associated lymphatic tissue (GALT)-related cells^3^,^7^,^8^,^9^,^10^. Human babies and newborns of other mammals enjoy the full benefits of Lf with milk consumption that is essential for their development. As they grow up, the maturation of the digestive system with age results in complete Lf digestion^1^. Lf breaks down into several large fragments by the gastric digestive enzyme, pepsin, but in case of direct intra-duodenal administration it may reach intestine and exist there for a few hours^3^. However, only the intact native Lf may reach the GALT and enter the lymphatic system, which is necessary for efficacy.

In this regard, there is a high demand for an appropriate Lf delivery system that should protect Lf from digestion in stomach and facilitate its permeability across intestinal epithelium. During the past decade, several oral Lf delivery systems have been suggested^2^,^3^,^4^,^5^,^6^,^7^,^8^,^11^,^12^,^13^,^14^,^15^. Nojima *et al*. first synthesized Lf conjugated with branched 20 kDa poly(ethylene glycol) (PEG) which demonstrated a significantly higher resistance to pepsin proteolysis in the mature rats^13^. The proteolytic half-life of the PEGylated Lf was found to be two times longer and its absorption from the rat intestinal tract increased ten times compared to unmodified Lf. Liposomes have also been used for Lf encapsulation^7^,^10^,^11^,^12^,^15^. Yamano *et al*. demonstrated that Lf encapsulated in liposomes had better resistance to digestive enzymes, thus enhancing the inhibitory effect of orally administered Lf on alveolar bone resorption using lipopolysaccharide-induced periodontitis rat models^10^. Onishi *et al*. developed a chitosan/alginate/Ca- microparticles, which were sufficiently small to enter the mucous layer of intestine^5^. Lf encapsulated in those microparticles showed better anti-inflammatory properties compared to free lactoferrin in a rat model with induced edema. However, the major drawbacks of all these systems are the use of organic solvents and harsh encapsulation conditions that may result in a significant loss of Lf bioactivities.

Layer-by-Layer (LbL) assembled microcapsules have been widely used for various applications as they provide high versatility with respect to payload and offer various targeted delivery and triggered release options, such as light, magnetic field, ultrasound, temperature, pH, salinity, redox potential^16^,^17^,^18^,^19^,^20^,^21^. This technology is specifically advantageous for encapsulation of fragile cargo in biomedical applications^22^ as it requires aqueous solutions only and is performed at room temperature. Moreover capsules may possess additional protective function^23^. Enzymatic cleavage is the release mechanism particularly important for these applications. Enzymatically degradable microcapsules have been extensively investigated during the last decade^24^,^25^,^26^,^27^,^28^,^29^,^30^,^31^,^32^,^33^ and employed for delivery of genes^24^,^26^, growth factors^29^,^30^ and vaccines^31^,^32^,^33^. The main drawback of all these microcapsules was the use of expensive and cytotoxic polycations. Capsules based on polysaccharides^34^ or inorganic clays^35^,^36^ were suggested to overcome this challenge. Recently, we reported LbL-assembled multilayer microcapsules made of low-cost and food grade ingredients (tannic acid (TA) and bovine serum albumin (BSA)) exploiting the ability of tannins to precipitate proteins by hydrogen bonding and hydrophobic interactions^37^,^38^. The microcapsules demonstrate low cytotoxicity, they are resistant towards treatment with trypsin but susceptible to α-chymotrypsin, the two proteolytic enzymes with different cleavage site specificity^38^. Incorporation of TA into the shell has an additional benefit providing the microcapsules with anti-oxidant properties by scavenging Fe^2+^ ions^39^,^40^.

This work is the first demonstration of the LbL-assembled BSA-TA microcapsules as a system for encapsulation and oral delivery of Lf. Here we make a special effort to maximize efficiency of Lf loading into the microcapsules while minimizing possible damage to its structure as proved by high performance liquid chromatography (HPLC). We show behavior of the microcapsules under simulated gastrointestinal conditions. In order to evaluate efficiency of proteins protection by microcapsules in gastric environment we encapsulate DQ™ Red BSA instead of Lf as digestion of this protein may be easily followed by fluorescence spectroscopy. Finally, we use NIR luminescence to follow the transit of Lf-containing microcapsules along the gastrointestinal tract (GIT) of mice *in vivo*, and Lf biodistribution in different parts of the mouse GIT and in liver *ex vivo*.

## Results and Discussion

### Lactoferrin encapsulation efficiency

**Figure 1:**
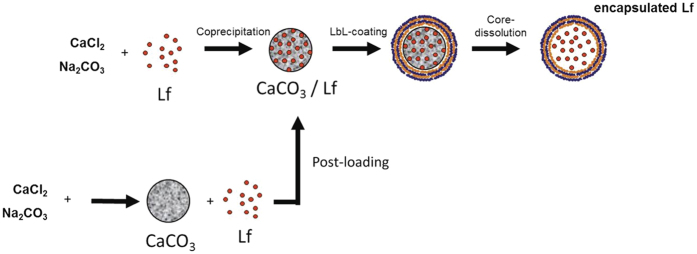
Scheme of Lactoferrin (Lf) encapsulation: co-precipitation of CaCO_3_ and Lf vs post-loading of Lf in porous CaCO_3_ microparticles both followed by Layer-by-Layer deposition of bovine serum albumin and tannic acid and final dissolution of CaCO_3_.

In this work, encapsulation of Lf was performed through its absorption by porous CaCO_3_ microparticles (corresponding SEM images are shown in the Supplementary Fig. S1), as first suggested by Volodkin *et al*.^41^. This material requires cheap precursors, is easy to synthesize, non-toxic and approved for use as additive in foods by FDA, Food Safety Commission in Japan, and in many other countries. Here we have tried two different approaches to load CaCO_3_ with Lf, as depicted in Fig. 1. In the first approach, formation of microparticles occurred in 30 mg/mL Lf solution, while in the second CaCO_3_ particles were first formed, washed and then redispersed in 30 mg/mL Lf aqueous solution. To measure the amount of absorbed Lf shown in Table 1, the particles were washed two times in DI water and then dissolved in HCl. Released Lf was quantified by ELISA and HPLC (for calibration curve and respective chromatograms see Supplementary Figs S2 and S3).

**Figure 2:**
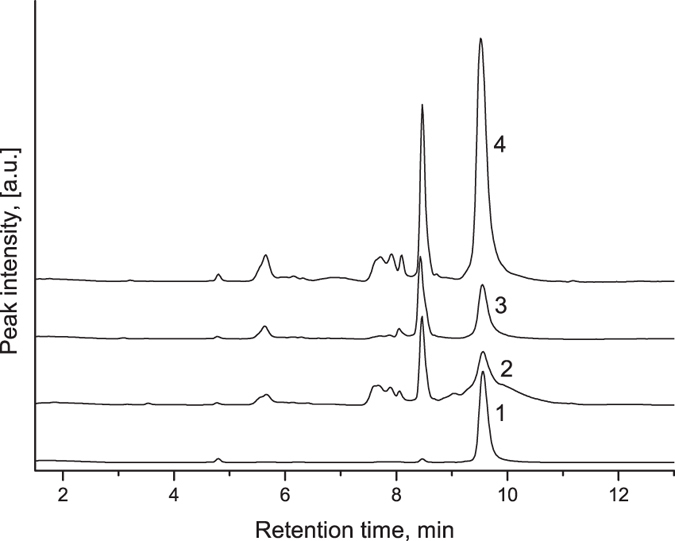
HPLC chromatograms of (1) a standard Lf (1 mg) solution in DI water, and Lf released from CaCO_3_ microparticles upon their dissolution at pH 3. Lf was (2) co-precipitated with 48 mg CaCO_3_ at pH 8; post-loaded to (3) 60 and (4) 300 mg of CaCO_3_. Volume of all samples was 6 mL.

The lowest amount of released Lf was achieved from samples prepared via the co-precipitation approach, when stoichiometric amounts (6×10^−4^ moles) of CaCl_2_ and Na_2_CO_3_ were mixed in the presence of Lf macromolecules (note that concentration of released Lf was too low for HPLC measurements and we used ELISA here). The reason could be significant Lf degradation upon synthesis due to high pH value in the resulted dispersion (pH > 10) caused by the injection of highly alkaline sodium carbonate solution. In fact, Lf treatment with 1 M sodium carbonate resulted in complete degradation of the protein (see corresponding HPLC chromatograms in Supplementary Fig. S4). In order to reduce the degradation of Lf, pH of the reaction mixture was decreased from >10.0 to 8.0 by increasing the ratio of CaCl_2_ concentration to that of Na_2_CO_3_ from 1.0:1.0 to 1.5:1.0. In these conditions, more than three times higher amount of Lf was released from CaCO_3_ particles as measured by ELISA. The chromatogram of this sample (see Fig. 2, line 2) demonstrates peak at retention time of 9.5 min characteristic for Lf but its full width at half height (FWHH) was 0.33 min, nearly two times wider if compared to native Lf (see Fig. 2, line 1, FWHH 0.17 min). Thus HPLC may be used here for qualitative analysis only. In addition, several more intense peaks appear at the lower retention times probably due to partial breakdown of Lf into smaller fragments during co-precipitation.

To overcome this problem, we suggest post-loading approach where CaCO_3_ particles were formed first and washed two times with DI water to remove all salts and decrease pH. These particles were later introduced to a solution of Lf. The chromatogram of released Lf (Fig. 2, line 3) demonstrates narrow peak at 9.5 min (FWHH 0.17 min, same as in native Lf) and another narrow peak of comparable intensity at a lower retention time of 8.3 min. Thus Lf still breaks down upon absorption by the particles at post loading, however, degradation is significantly lower than in co-precipitation approach. Taking into consideration the importance of native Lf for bioavailability, post-loading approach was chosen for further experiments.

The weight percentage of absorbed Lf was nearly constant at ~1.2–1.4 wt. % when 5 and 10 times more CaCO_3_ microparticles were introduced into the Lf solution (see Table 1). This means that the chosen concentration (30 mg/mL) was adequate to ensure saturation of all microparticles with Lf to their maximum capacity.

**Figure 3:**
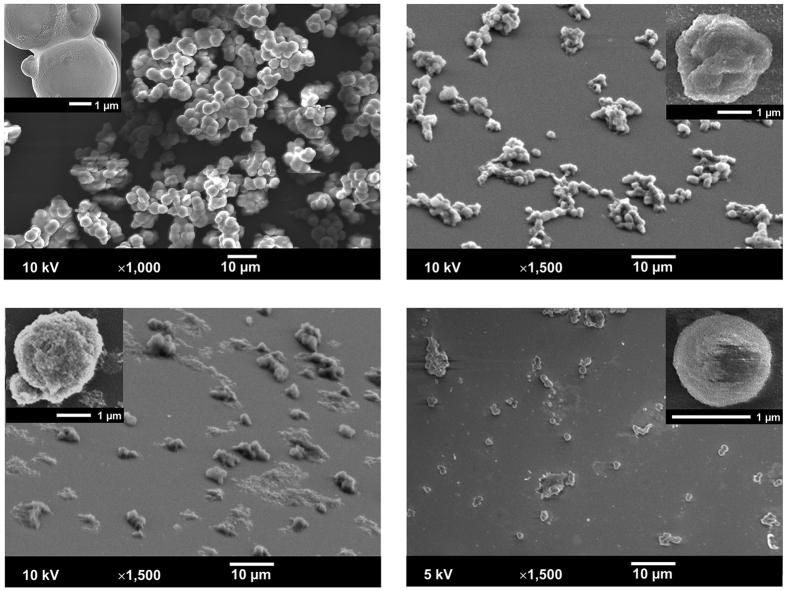
SEM images of (**a**) Lf/CaCO_3_ microparticles coated with (BSA-TA)_4_ multilayer shell; (**b**) microcapsules after CaCO_3_ core dissolution; (**c**) after 60 min of their treatment in simulated gastric fluid and (**d**) after 3 min of treatment in simulated intestine fluid.

In the next step, LbL assembly of the BSA-TA shells was performed on the surface of CaCO_3_/Lf particles followed by dissolution of the core material with HCl thus making Lf-loaded BSA-TA microcapsules. Corresponding SEM images are shown in Fig. 3-a,b. The microcapsules have smaller size and much rougher surface compared to the coated microparticles but their shape is still close to spherical.

In order to measure the amount of Lf in the final microcapsules, they were treated with 8 M urea solution. Under these conditions the microcapsules were disintegrated (see corresponding CSLM images in Supplementary Fig. S5) and all the proteins (BSA, Lf) underwent denaturation. The resulted solution has been tested by western blot (see Supplementary Fig. S6). The microcapsules fabricated from 300 mg CaCO_3_ microparticles by the post-loading approach contained 0.72 mg of encapsulated Lf and 6 mg of total proteins (including BSA and Lf, both intact and fragmented). If compared to 4.1 mg of Lf absorbed in CaCO_3_ microparticles (see Table 1), one can see that ~18% of initially encapsulated Lf retained inside upon LbL assembly and dissolution of the core and the rest was lost.

HPLC and western blot show the total amount of encapsulated Lf in a batch but provide no information about its amount in an individual microcapsule and the distribution of Lf among the microcapsules in the batch. Lf has a high affinity to Fe^3+^ and is believed to play a major iron-regulating role in new born infants and mammals^1^. Since Fe gives high ionization yield and ToF-SIMS provides submicron spatial resolution, it is possible to detect amount of Lf in individual microcapsules^42^. The C^+^ and Fe^+^ maps and corresponding distribution of Lf content among microcapsules are shown in Supplementary Fig. S7. The distribution can be well fitted with a Gauss curve, where the mean Fe/C ratio (R) was found to be 0.54 ± 0.16, and the polydispersity index or span defined as (R90 – R10)/R50 = 0.92.

### *In vitro* digestion study of encapsulated Lf

The performance of encapsulated Lf in GIT was evaluated using *in vitro* simulated digestive model^43^. Table 2 shows the weight and corresponding weight loss of empty and Lf-loaded (BSA-TA)_4_ microcapsules, as prepared and upon simulated digestion.

As Table 2 shows, treatment with HCl at pH 3 dissolves all CaCO_3_ (~300 mg) from the microcapsules’ cores. The microcapsules loaded with Lf are nearly two times heavier than empty microcapsules that may be an indication of the amount of both intact Lf and its fragments in the microcapsules. However, the possible effect of Lf absorbed by CaCO_3_ particles on the later process of BSA-TA shell assembly on their surface making it thicker and/or denser could not be excluded. The SEM shows that the Lf-loaded microcapsules have spherical shape while the microcapsules without Lf are partially collapsed (compare Fig. 3-b and Supplementary Fig. S8).

The Lf-loaded microcapsules remain stable in SGF losing only 5% of their weight after 60 min of treatment. SEM also does not show significant changes in the microcapsules size or shell integrity (see Fig. 3-c). However, fast degradation of the microcapsules occurs in SIF demonstrating loss of ~70% of initial weight after 3 min and nearly complete degradation after 10 min of treatment. SEM image of microcapsules treated in SIF for 3 min demonstrate less number of microcapsules as shown in Fig. 3-d. The fast degradation of BSA-TA microcapsules in SIF releasing the payload was also confirmed by CLSM for the microcapsules loaded with BSA/FITC (see Supplementary Fig. S5).

The reason for such drastic changes in shells’ stability could be different cleavage site specificity of pepsin and proteases of pancreatin (trypsin and chymotrypsin). Pepsin preferentially cleaves peptide amide bonds of large hydrophobic amino acids (tyrosine, tryptophan, or phenylalanine) and BSA contains only 6.56% of phenylalanine, 5.06% of tyrosine, and 0.58% of tryptophan^44^. Chymotrypsin is targeting the same amino acids as pepsin while trypsin cleaves peptide chains mainly at the carboxyl side of the amino acids lysine or arginine and their total amount in BSA is rather high (18.72%^44^).

**Figure 4:**
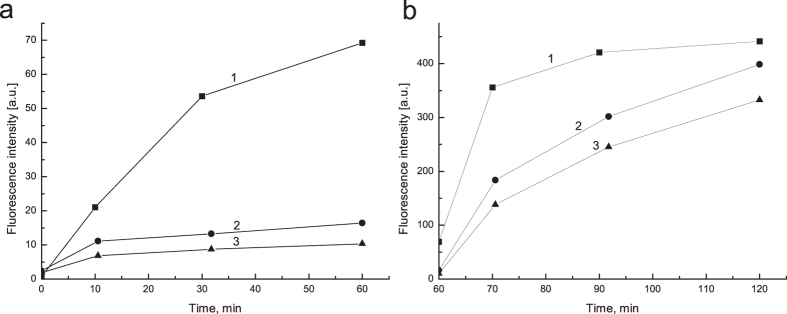
Fluorescence intensities with respect to (**a**) digestion time in SGF followed by (**b**) digestion time in SIF of (1) free DQ™ Red BSA solution obtained upon post-loading the CaCO_3_ microparticles with protein followed by their dissolution at pH 3; DQ™ Red BSA encapsulated by post-loading in (2) (BSA-TA)_4_ shells and (3) (BSA-TA)_8_ shells.

In order to check whether BSA-TA shells provide protection for encapsulated proteins against gastric digestion, DQ™ Red BSA was encapsulated instead of Lf. This protein is heavily labeled with BODIPY dyes and, when in intact native confirmation, it doesn’t show fluorescence due to strong quenching effect. This quenching is relieved upon protein cleavage by proteases. In order to match concentration of DQ™ Red BSA, the sample with free unprotected protein was prepared as the following. First, DQ™ Red BSA was loaded into CaCO_3_ microparticles by the same post-loading approach as for microcapsules preparation, then the particles were dissolved by acidifying the solution to pH 3 with HCl and the resulted solution was exposed to SGF. The fluorescent intensity was increased during 60 min of treatment due to gradual digestion of the protein, as shown in Fig. 4-a (line 1). Contrary, the samples with DQ™ Red BSA encapsulated in (BSA-TA)_4_ and (BSA-TA)_8_ shells demonstrated some increase in fluorescent intensity within first 10 min of SGF digestion only, and fluorescence nearly didn’t increase upon further treatment (Fig. 4-a, lines 2, 3). This initial increase in fluorescence intensity could be a result of some defect loose shells within a batch that allow pepsin to penetrate inside. While the number of layers increases the defects are lesser and the amount of such loose shells decreases providing better protection.

We introduce a protective efficiency parameter, P.E. = (1 − I_x_/I_0_) × 100%, where I_x_ is a fluorescence intensity of DQ™ Red BSA encapsulated in (BSA-TA)_4_ or (BSA-TA)_8_ shells, and I_0_ is the fluorescence intensity of unprotected protein at the same point of digestion time. After 60 min of treatment, P.E. was found to be 76 ± 6% and 85 ± 2% for (BSA-TA)_4_ and (BSA-TA)_8_, correspondingly.

In the *in-vitro* digestive model used in this work, intestinal digestion starts after 60 min of gastric digestion. Figure 4-b shows corresponding changes in fluorescent intensity. In the sample with free unprotected DQ™ Red BSA, fluorescence increases dramatically from 60 to 70 min of digestion (within first 10 min of SIF treatment) and then reaches the plateau indicating complete digestion of introduced DQ™ Red BSA by this point of time. As it was discussed above, BSA is much more susceptible to proteases of pancreatin than to pepsin, so significantly higher fluorescent levels in SIF are expected.

In the sample with encapsulated DQ™ Red BSA, the fluorescence intensity increases gradually from 60 to 120 min of digestion. As it was shown in Table 2, BSA-TA shells degrade nearly completely within 10 min in SIF. However digestion of encapsulated DQ™ Red BSA is delayed significantly. The reason could be some aging of pancreatin enzymes as they are involved in additional degradation cycles of both the shell and encapsulated protein. Nevertheless, the fluorescence intensity of DQ™ Red BSA encapsulated in (BSA-TA)_4_ and (BSA-TA)_8_ shells by the end of 120 min of digestion was lower by just 10 and 25%, respectively, when compared to unprotected protein. This demonstrates nearly complete release of encapsulated proteins from the microcapsules as they reach an intestine.

### *In vivo* and *ex vivo* analysis of Lf progression along mice GIT and accumulation in liver

**Figure 5:**
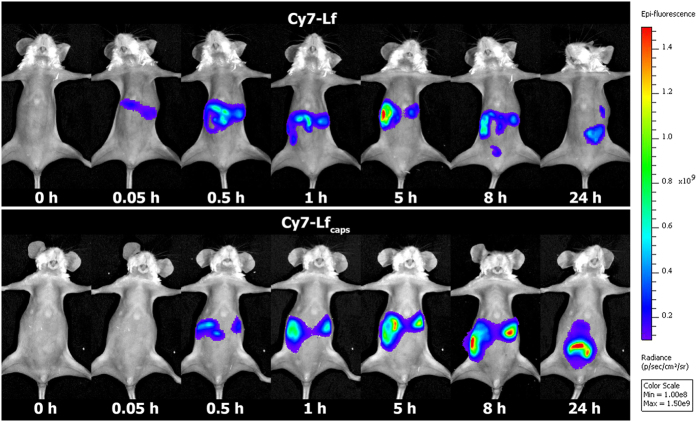
Mouse whole body images and corresponding Cy7 signal at 0.05, 0.5, 1, 5, 8 and 24 h after dosing with Cy7-Lf, and Cy7-Lf_caps_.

In order to track progression of encapsulated Lf along GIT *in vivo* we have used Cy7-Lf which emits fluorescence in the NIR region (λ_em_ = 800 nm). For small animals, ratio of this signal to nose near the skin is high enough allowing direct and non-invasive tracking of microcapsules^45^. Figure 5 shows progression of the dye for mice dosed with Cy7-Lf and encapsulated Cy7-Lf (Cy7-Lf_caps_). In order to match amount of Cy7-Lf_caps_, the sample with free protein was prepared by post-loading of Cy7-Lf into 300 mg of freshly prepared CaCO_3_ microparticles followed by their dissolution with HCl till pH 3.

At 0.05 h after administration, fluorescence has been detected in stomach of mice dosed with Cy7-Lf, while it was comparable to background in those dosed with Cy7-Lf_caps_. At 1 h, fluorescence was registered in stomach and the proximal part of the small intestine, and at 8 h, the chymus reached large intestine for both groups. At this time point, the mice dosed with Cy7-Lf_encaps_ had very high fluorescence in the distal parts of small intestine. One possible reason could be that this part of the mouse GIT is localized closer to skin so that the optical signal is less damped. However the mice dosed with Cy7-Lf had much lower fluorescence there. At 24 h, fluorescence was registered in the distal parts of small intestine and in large intestine for both groups, although for mice dosed with Cy7-Lf_encaps_ it was significantly higher.

**Figure 6:**
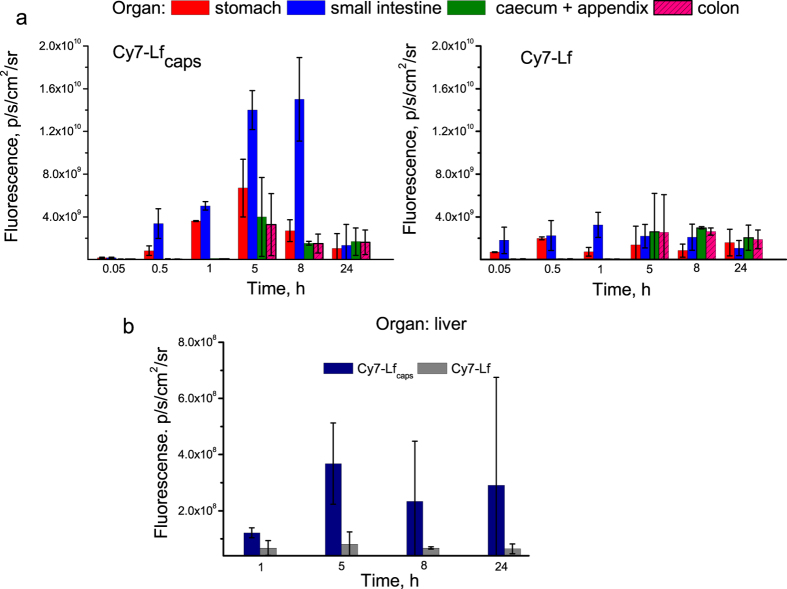
(**a**) Fluorescence intensity in mouse stomach, small intestine, caecum/appendix, and colon at 0.05, 0.5, 1, 5, 8, and 24 h after dosing with Cy7-Lf_caps_ and Cy7-Lf. (**b**) Fluorescence intensity in mouse liver at 1, 5, 8, and 24 h after dosing with Cy7-Lf_caps_ and Cy7-Lf.

Figure 6-a shows fluorescence intensities in mouse stomach, small intestine, caecum/appendix and colon for both groups of mice. In the time span from 0.05 to 1 h, fluorescence was registered in stomach and small intestine only. At 0.05 h, it was much lower for the group of mice dosed with Cy7-Lf_caps_, but from 0.5 h onwards this group had much higher fluorescence intensity, especially in the small intestine. For example, at 5 h time point it was 6.5 times higher than in the control group. From 5 h onwards, fluorescence was registered at caecum/appendix and colon.

Very low fluorescence of Cy7-Lf_caps_ in the initial digestion phase (0.05 h) could be attributed to a self-quenching effect due to high local concentration of dye in the intact microcapsules. Once the microcapsules reach small intestine they are digested releasing Lf that is diluted in the chymus. As a result fluorescence signal increases dramatically (see Figs 5 and 6-a).

Released Lf and its digested fragments are absorbed into the bloodstream. The level of this absorption may be estimated using the intensity of fluorescence in mouse liver, shown in the Fig. 6-b. For mice dosed with Cy7-Lf_caps_, this intensity increases from 1·10^8^ to 4·10^8^ p/s/cm^2^/sr in the time span from 1 to 5 h post-administration and then remains relatively stable over 24 h. However for mice in the control group dosed with Cy7-Lf, the liver fluorescence was always comparable to the autofluorescence level of (2–7)·10^7^ p/s/cm^2^/sr. Thus encapsulation in BSA/TA shells ensures detectable levels of Lf in the blood stream.

## Conclusion

In this work for the first time we introduce the LbL-assembled BSA-TA microcapsules as a system for oral delivery of Lf. Its efficiency is demonstrated using *in vitro* digestion model, *in vivo* small animal model and *ex vivo* analysis. Absorption by freshly prepared porous CaCO_3_ microparticles followed by LbL assembly of the BSA-TA shell and dissolution of the cores was suggested as the less harmful Lf loading method. One batch of the microcapsules contains 0.72 mg of native Lf. The Lf distribution among the microcapsules was normal with a span of 0.92 underpinning good capsule loading over population of microcapsules in the sample. The microcapsules showed high stability in simulated gastric conditions and effective protection of encapsulated proteins (DQ™ Red BSA) from gastric digestion. Protective efficiency was found to be 76 ± 6% and 85 ± 2%, for (BSA-TA)_4_ and (BSA-TA)_8_ shells, correspondingly. The shells are degraded in the simulated intestinal conditions releasing the proteins at their beneficial site of action. The encapsulated Lf is released in small intestine where it is absorbed into the bloodstream demonstrating detectable levels of Lf in liver. At the same time it could happen that simple Lf release in the intestine might not be enough to achieve its full biological action and more specific targeting to the receptors of GALT might be required. This may be achieved by additional incorporation of gastro-adhesive proteins into the shells and these experiments are currently in progress in our laboratory.

We believe that suggested encapsulation system has a great potential for oral delivery of a number of various active food ingredients beyond lactoferrin, including other bioactive proteins, prebiotics, probiotics, fatty acids, natural inhibitors of glycoside hydrolases, and many other ingredients that require protection from gastric digestion and site-specific release in intestine to achieve their full function. In a near future we may expect a progress in this area and the development of foods that will not only provide people with nutrients but stimulate their immune system, improve health and lifestyle.

## Materials and Methods

### Materials

The bioactive Lf from bovine whey was kindly supplied by the Tatua Cooperative Dairy Company Ltd (New Zealand). Poly-L-Lysine hydrochloride (PLL, Mw 15,000–30,000), BSA lyophilized powder (≥96%), TA (ACS grade), BSA conjugated with fluorescein isothiocyanate (BSA/FITC), bile salts (for microbiology), pancreatin from porcine pancreas (≥100 USP U/mg), pepsin from porcine gastric mucosa (3802 U/mg), mini protease inhibitor cocktail cOmplete™, hydrochloric acid, calcium chloride dehydrate, sodium bicarbonate, sodium chloride, sodium hydroxide, acetonitrile (for HPLC, ≥99.9%) were purchased from Sigma-Aldrich. Anhydrous sodium carbonate was purchased from Alfa Aesar. 0.1% aqueous solution of trifluoroacetic acid (TFA, LC/MS grade, ≥99.99%) was purchased from VWR Chemicals. DQ™ Red BSA was purchased from Molecular Probes Inc, USA. The bovine lactoferrin ELISA kit was purchased from Bethyl Laboratories, Inc., USA. Cyanine 7 N-hydroxysuccinimide ester (Cy7-NHS) was purchased from Lumiprobe Corporation, USA. Cy7-labelled Lf (Cy7-Lf) has been prepared according to a standard protocol provided by Lumiprobe Co and purified by dialysis. All chemicals were used as received without further purification. Deionized (DI) water (specific resistivity higher than 18.2 MΩcm) from Milli-Q plus 185 (Millipore) water purification system was used to make all solutions.

### Preparation of microcapsules loaded with proteins

BSA-TA microcapsules loaded with proteins (Lf, Cy7-Lf, BSA-FITC or DQ™ Red BSA) were prepared with assistance of a CaCO_3_ sacrificial template, as depicted in Fig. 1. Spherical porous CaCO_3_ particles with an average diameter of ~3 μm were synthesized according to Volodkin *et al*.^41^. In post-loading, 0.6 mL of 1 M CaCl_2_ and Na_2_CO_3_ solutions were injected into 1.8 mL of DI water under vigorous agitation. 2 min later agitation was stopped and CaCO_3_ particles were separated by centrifugation and washed two times with DI water. Subsequently, the particles were re-dispersed in 30 mg/mL Lf aqueous solution and shaken for 15 min. In co-precipitation, 1.8 mL of an aqueous protein solution (30 mg/mL Lf or 1 mg/mL DQ™ Red BSA) was mixed with 1 M CaCl_2_ solution (0.6–0.8 mL) followed by injection of 1 M Na_2_CO_3_ solution (0.4–0.6 mL) under vigorous agitation.

LbL assembly of BSA-TA shells was performed as the following. First, CaCO_3_ microparticles with absorbed proteins were separated by centrifugation, washed two times with DI water and immersed in 5 ml of 2 mg/mL PLL solution in order to generate the first anchoring layer. After 15 min of continuous shaking, the microparticles were collected by centrifugation and residual PLL was removed by washing them twice with DI water. Further alternating layers of BSA and TA were introduced, each from 5 mL of 2 mg/mL solution with two washing steps after each layer. This procedure was repeated to achieve desired number of BSA-TA bilayers. Finally, the particles were redispersed in DI water and CaCO_3_ was dissolved by adding 1 M HCl solution dropwise until pH reaches ~3.0. The resulted microcapsules were collected by centrifugation and washed two times with DI water.

### Microcapsules behavior under simulated gastrointestinal conditions

Here we used the following *in vitro* digestion protocol^43^. All experiments were done at 37 °C under continuous agitation. 4 mL of 150 mM NaCl solution at pH 3 (acidified by HCl) was added to 5 mL of a suspension containing ~ 1 mg of encapsulated protein. Then 1 mL of 7.1 mg/mL pepsin solution containing 150 mM of NaCl at pH 7.0 was injected there (pepsin final concentration was 180 Units per 1 mg of a protein). This was considered as a starting point for digestion. At 60 min of digestion, pH of the solution was increased to 7.0, 1 mL of 120 mM bile salts (containing 0.1 M NaHCO_3_ solution at pH 7.0) and 1.0 ml of 18 mg/mL pancreatin solution (pancreatin final concentration was 12 USP U/mg) were added. To stop digestion at a selected point of time, pH of the solution was increased to 7.0 (with a predetermined amount of NaOH), a protease inhibitor tablet was added and the solution was frozen at −20 °C before further analysis. For weight loss experiments, all the samples were defrozen, the microcapsules were separated by centrifugation, washed two times with DI water and freeze-dried for 2 days using Console Freeze Dry System from Labconco.

### *In vivo* digestion and *ex vivo* Lf biodistribution studies

A total of sixteen BALB/c female mice were used in this study. All experiments were performed in accordance with relevant guidelines and regulations. Animal ethics clearance was approved by the ethical committee of National Research Ogarev Mordovia State University, Russia. The abdomen of each animal was carefully shaved to improve fluorescence acquisition. 0.3 mL of a suspension containing 4 mg of encapsulated Cy7-Lf was dosed directly into the stomach of eight mice *via* oral gavage. A control group of eight mice was dosed with solutions of free Cy7-Lf. Prior to imaging, animals were anaesthetized by intramuscular injection of zoletil (50 mg/kg) and placed in an imaging cradle. The mice were imaged at 0.05, 0.5, 1, 5, 8 and 24 h after dosing with an IVIS imaging system (Xenogen Corp.) using excitation/emission at 675/810-875 nm. Photons were quantified using LivingImage software (Xenogen Corp). At 0.05 and 0.5 h post-administration, Lf was mostly located within the stomach and other organs demonstrate autofluorescence only. Therefore, in order to save the animals, levels of fluorescence in stomach and proximal parts of small intestine were estimated from *in vivo* measurements using the corresponding damping coefficients determined for mice sacrificed at later time points. Two mice from each group have being euthanized by cervical dislocation for further *ex vivo* analysis at each time point in the range from 1 to 24 h. For all animals, the gastrointestinal tracts and liver were removed and imaged.

### Characterization

Concentration of released Lf in aqueous solutions was independently measured by bovine lactoferrin ELISA kit (Bethyl Laboratories, Inc., USA) following their standard protocol and by HPLC using a Waters 2695 Alliance System equipped with 2996 photo diode array detector. The column was Phenomenex Aeris XB-C8, particle diameter 3.6 μm WIDEPORE, 4.6 mm × 100 mm. Prior to analysis, all the samples were filtered through a 0.45 μm syringe filter, the injection volume of sample was 50 μL and detection wavelength was at 210 nm. A continuous gradient elution at 35 °C and 1.0 ml/min flow rate was performed with 0.1% aqueous TFA solution (mobile phase A) and 90% acetonitrile – 10% aqueous TFA solution (mobile phase B) as following: the percentage of the mobile phase B was increased linearly from 20 to 50% by 15 min, then decreased back to 20% by 20 min of elution. All of the experiments were done in triplicate. First, concentration of Lf in samples was determined using the corresponding calibration curve. Second, amount of Lf (in mg) was calculated for each sample using known volumes of the initial dispersion and of 1 M HCl solution required to bring pH value down to 3.0 (assuming all CaCO_3_ is dissolved at this pH). Quantification of lactoferrin incorporated into microcapsules was performed by western blot (see details in the Supplementary information).

Scanning electron microscopy (SEM) analysis was performed using field-emission scanning electron microscope (FE SEM JSM-6700F). Samples were prepared by depositing a drop of microparticles or microcapsules suspension on a silicon wafer allowed to dry at room temperature. Before imaging, the samples were coated with approximately 20 nm gold film using a Denton sputter-coater.

Confocal micrographs were taken with Olympus FluoView FV1000 (Olympus, Japan) laser scanning confocal microscope (CLSM) using a 100/1.45 oil objective. The excitation (λ_exc_) and emission (λ_em_) wavelengths λ_exc_ = 488 nm, λ_em_ = 525 nm were used for scanning of BSA/FITC.

Distribution of Lf among the BSA-TA microcapsules has been studied by Time-of-flight secondary ions mass spectroscopy (ToF-SIMS, see details in the Supplementary information).

The measurements of fluorescence intensity have been performed using PerkinElmer Luminescence Spectrometer LS 55. The emission spectra have been recorded over a range of 595–720 nm wavelengths; excitation wavelength was 590 nm, the emission slit widths was 10 nm, and the scanning speed was 50 nm/min. All measurements have been performed using disposable polystyrene cuvettes with optical path length of 10 mm at room temperature (22 °C). All the emission spectra registered had maximal intensity at 620 nm.

## Additional Information

**How to cite this article:** Kilic, E. *et al*. Formulation for Oral Delivery of Lactoferrin Based on Bovine Serum Albumin and Tannic Acid Multilayer Microcapsules. *Sci. Rep.* **7**, 44159; doi: 10.1038/srep44159 (2017).

**Publisher's note:** Springer Nature remains neutral with regard to jurisdictional claims in published maps and institutional affiliations.

## Supplementary Material

Supplementary Information

## Figures and Tables

**Table 1:** Amount of lactoferrin absorbed by CaCO_3_ particles.

Approach	Weight of CaCO_3_ microparticles, mg	Amount of released Lactoferrin, mg	Lactoferrin absorbed, wt%
Co-precipitation (pH > 10)	60	0.3 ± 0.1^a^	0.5 ± 0.2
Co-precipitation (pH 8)	48	1.0 ± 0.1^a^	2.1 ± 0.2
Post-loading	60	0.7 ± 0.1^b^	1.2 ± 0.2
	300	4.1 ± 1.5^b^	1.4 ± 0.5
	600	7.6 ± 2.0^b^	1.3 ± 0.3

Methods to measure Lf concentration: ^a^-ELISA; ^b^-HPLC.

**Table 2:** Weight and corresponding weight loss of Lf/(BSA-TA)_4_ microcapsules upon their treatment with simulated gastric (SGF) and intestine (SIF) fluids.

	Weight, mg	Weight loss, %
CaCO_3_/(BSA-TA)_4_	317.2 ± 6	NA
(BSA-TA)_4_	15 ± 2	NA
Lf/(BSA-TA)_4_	32.1 ± 3	0
SGF, 60 min	30.6 ± 1	5 ± 3
SIF, 3 min	9 ± 2	72 ± 6
SIF, 10 min	1.10 ± 0.08	96.6 ± 0.25
SIF, 60 min	0.9 ± 0.6	97.2 ± 2
