# Supplementary material for: Formulation for Oral Delivery of Lactoferrin Based on Bovine Serum Albumin and Tannic Acid Multilayer Microcapsules
Source: Sci Rep. 2017 Mar 10;7:44159. doi: 10.1038/srep44159 (PMC5344998; doi:10.1038/srep44159)
Supplement: Supplementary Information [file srep44159-s1.doc]

**Supplementary** **information**

Formulation for Oral Delivery of Lactoferrin Based on Bovine Serum Albumin and Tannic Acid Multilayer Microcapsules

Ece Kilic, Marina V. Novoselova, Su Hui Lim, Nikolay A. Pyataev, Sergey I. Pinyaev, Oleg A. Kulikov, Olga A. Sindeeva, Oksana A. Mayorova, Murney Regan, Maria N. Antipina, Brendan Haigh, Gleb B. Sukhorukov, Maxim V. Kiryukhin*

**
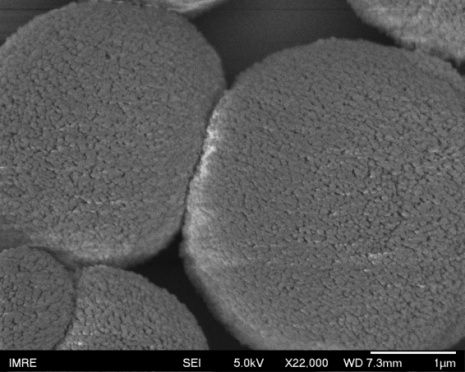
**

**
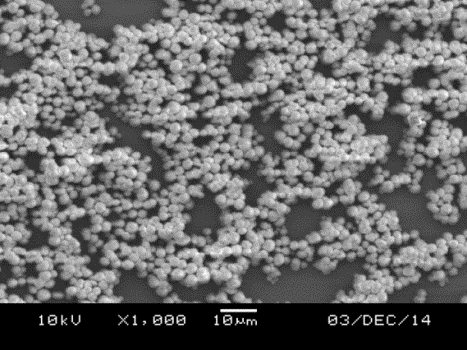
**

**Figure S1.** SEM images of CaCO_3_ microparticles. All the microparticles have spherical shape, are 2-4 μm in size and have very rough porous surface.


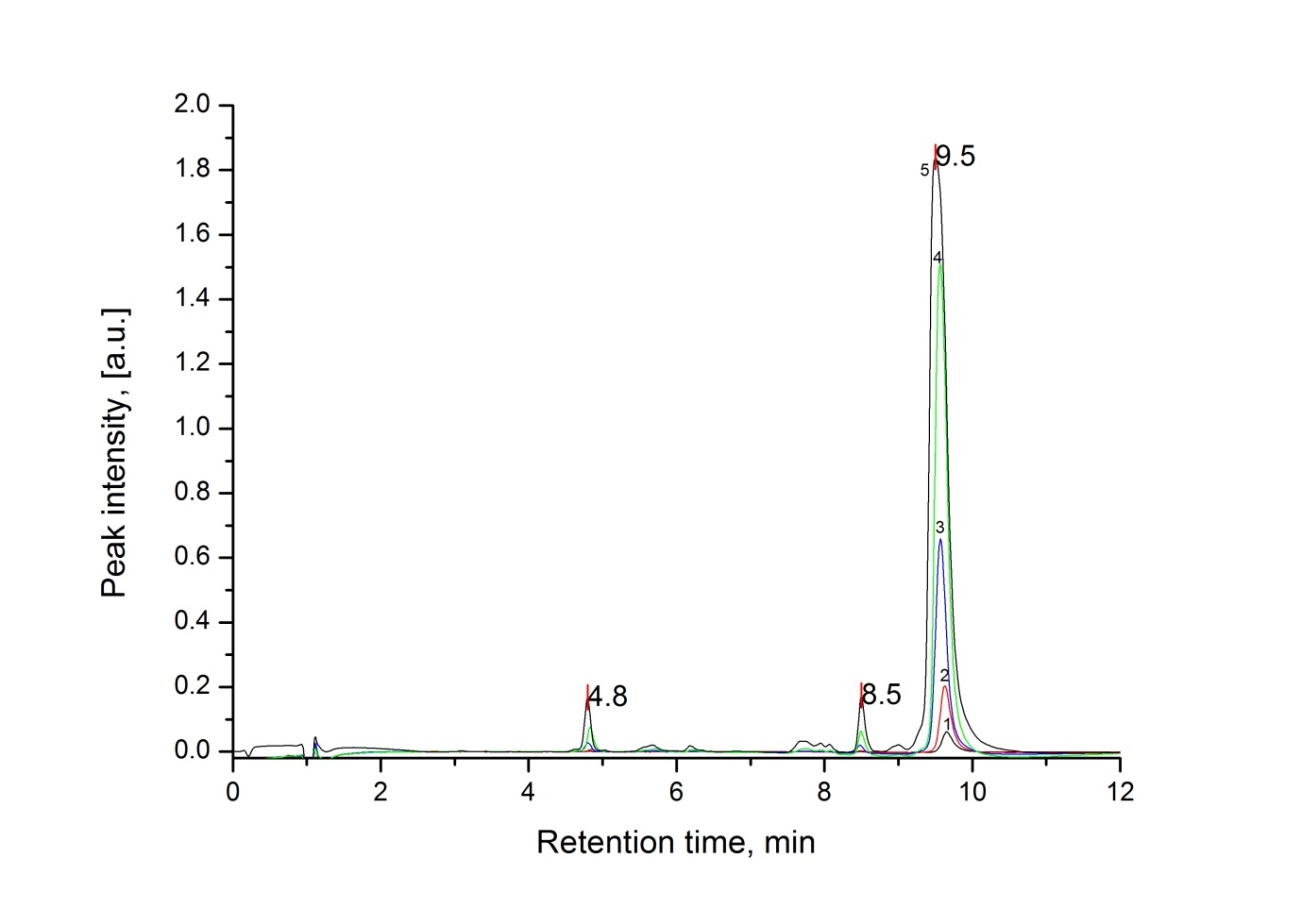


**Figure S2.** HPLC chromatograms of Lf standard solutions with concentrations 0.01 mg/mL (1), 0.06 mg/mL (2), 0.2 mg/mL (3), 0.5 mg/mL (4), and 1.0 mg/mL (5).


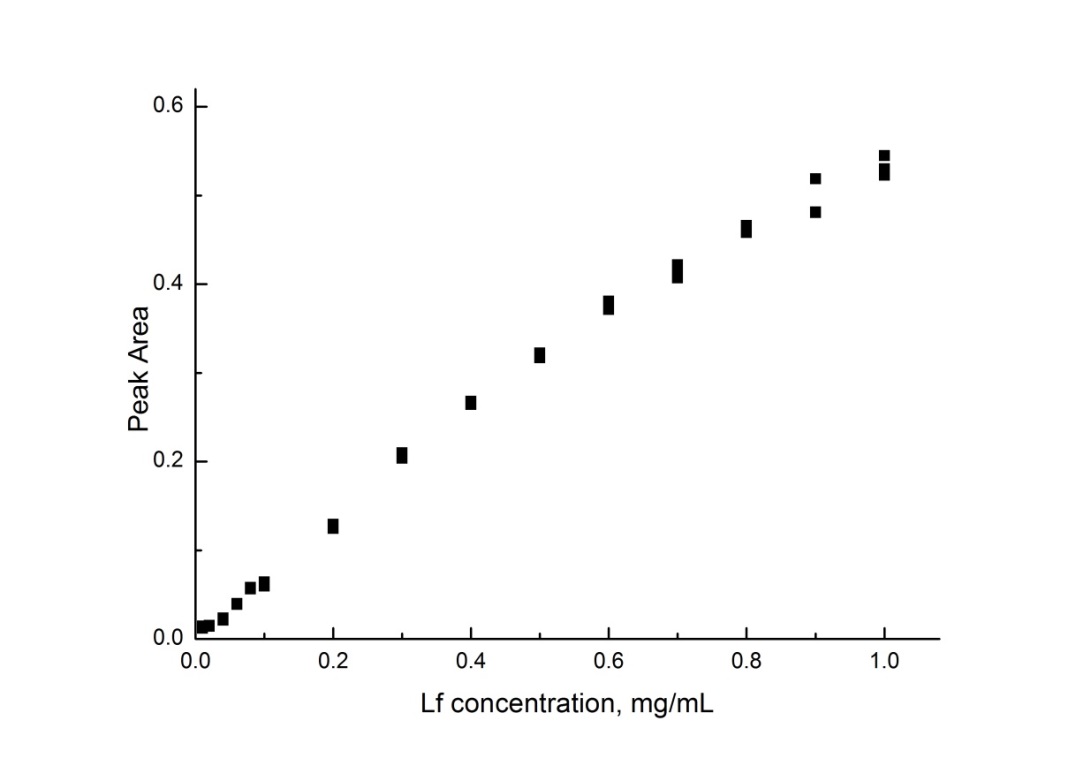


**Figure S3.** Calibration curve used to measure Lf concentration from the area under the peak at 9.5 min of retention time.


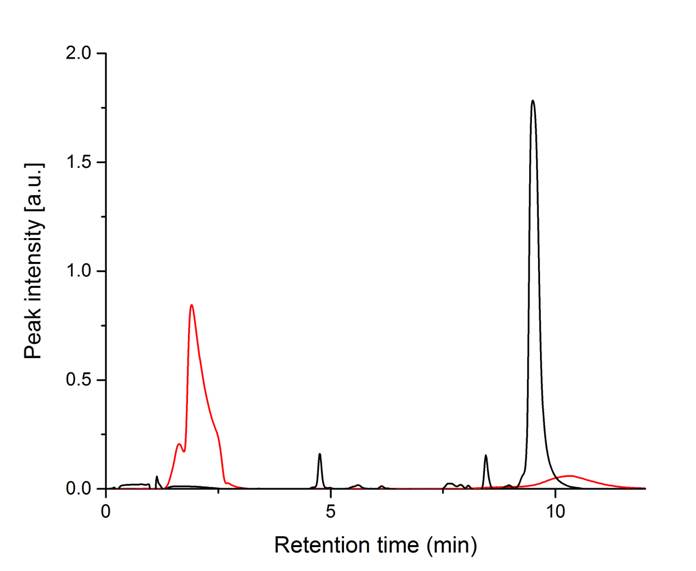


**Figure S4.** HPLC chromatograms of Lf before and after treatment with Na_2_CO_3_, 1M.


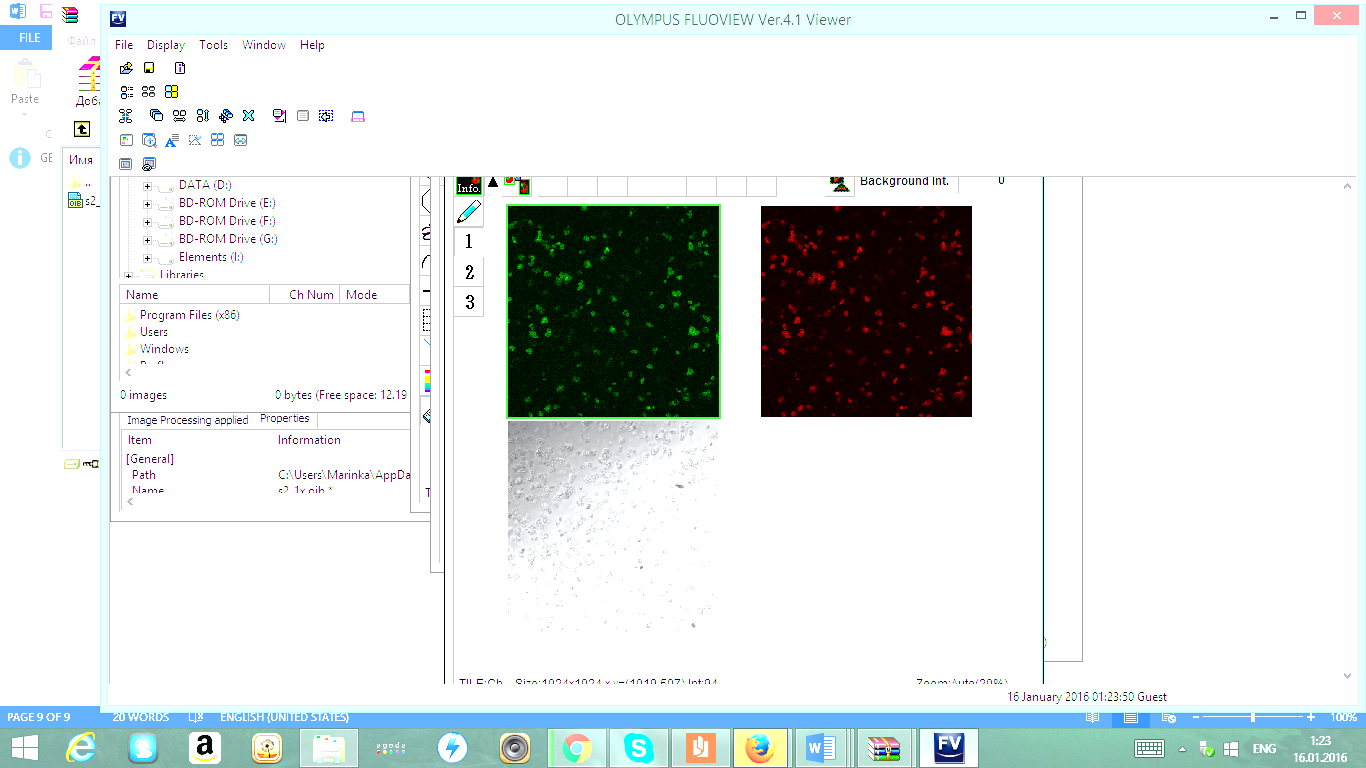

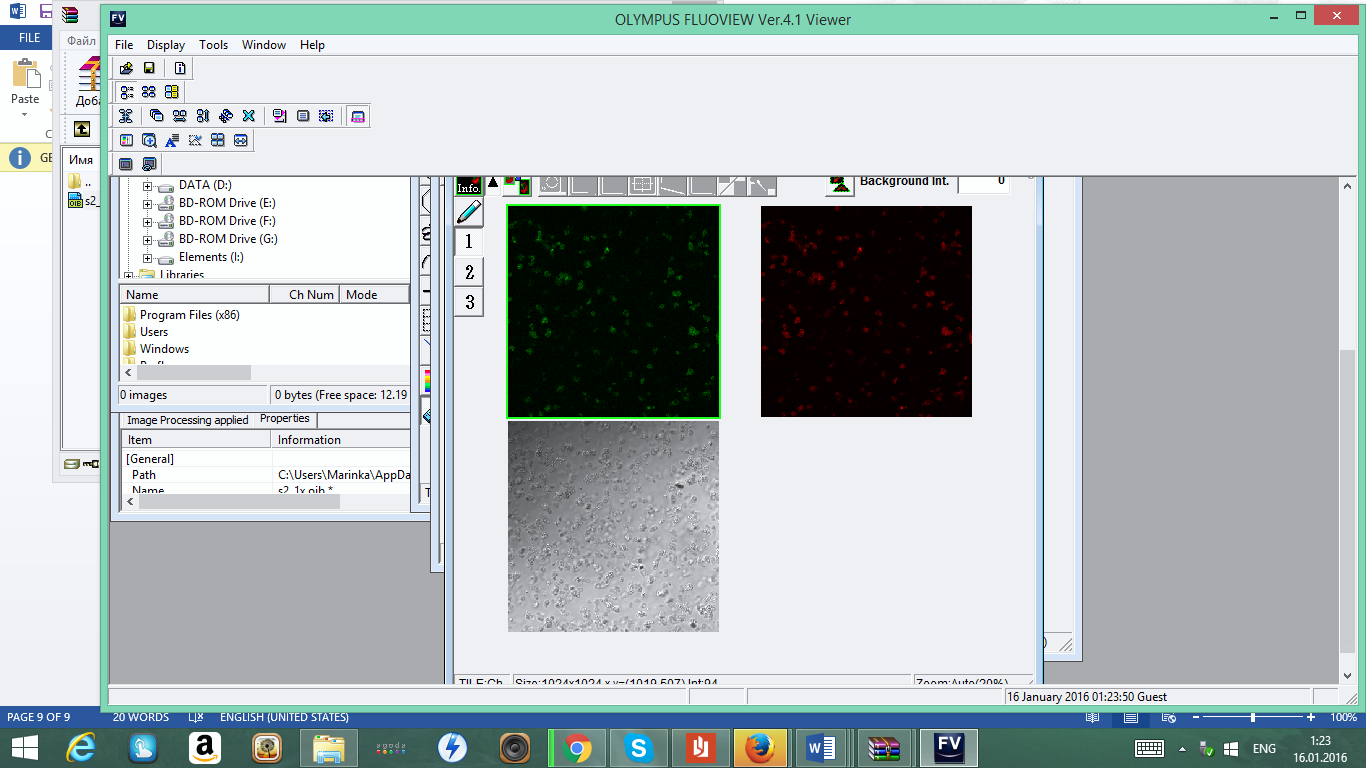


**(a)**


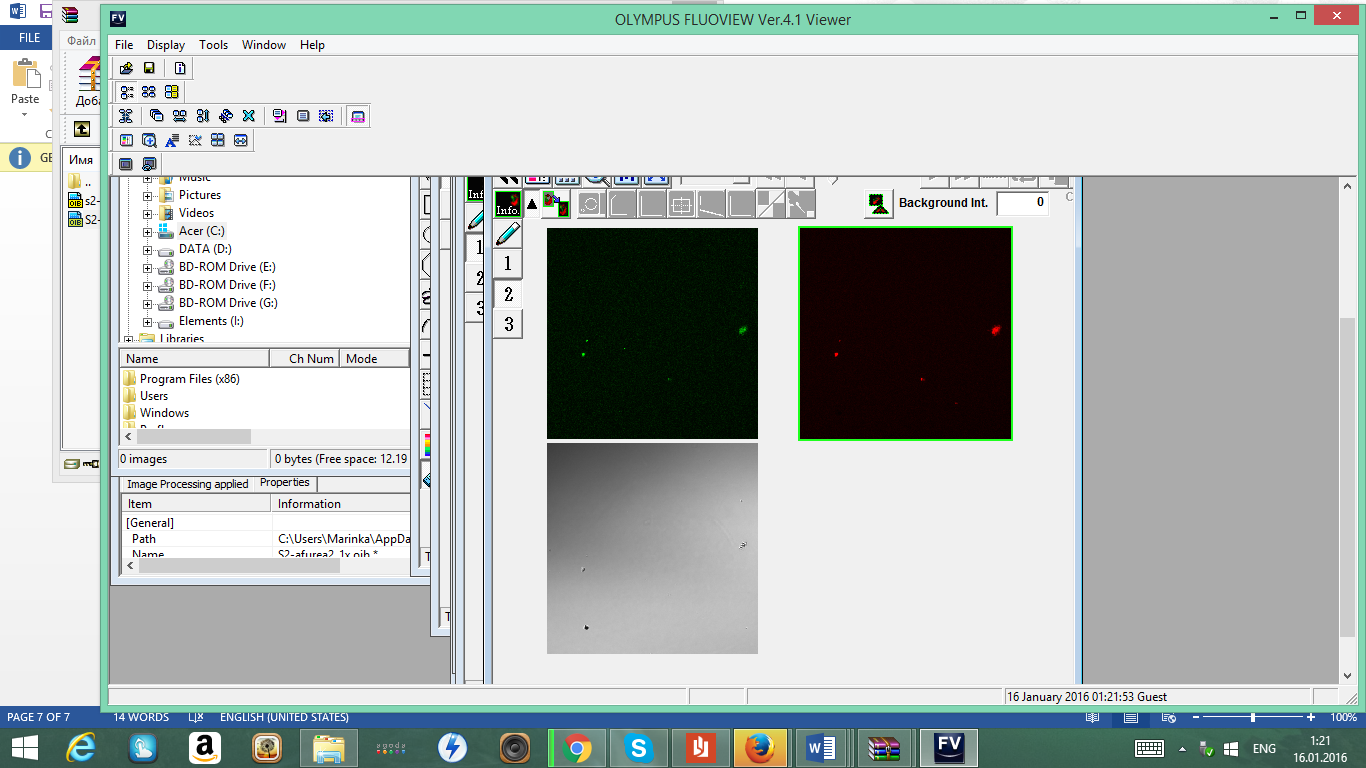


**(b)**


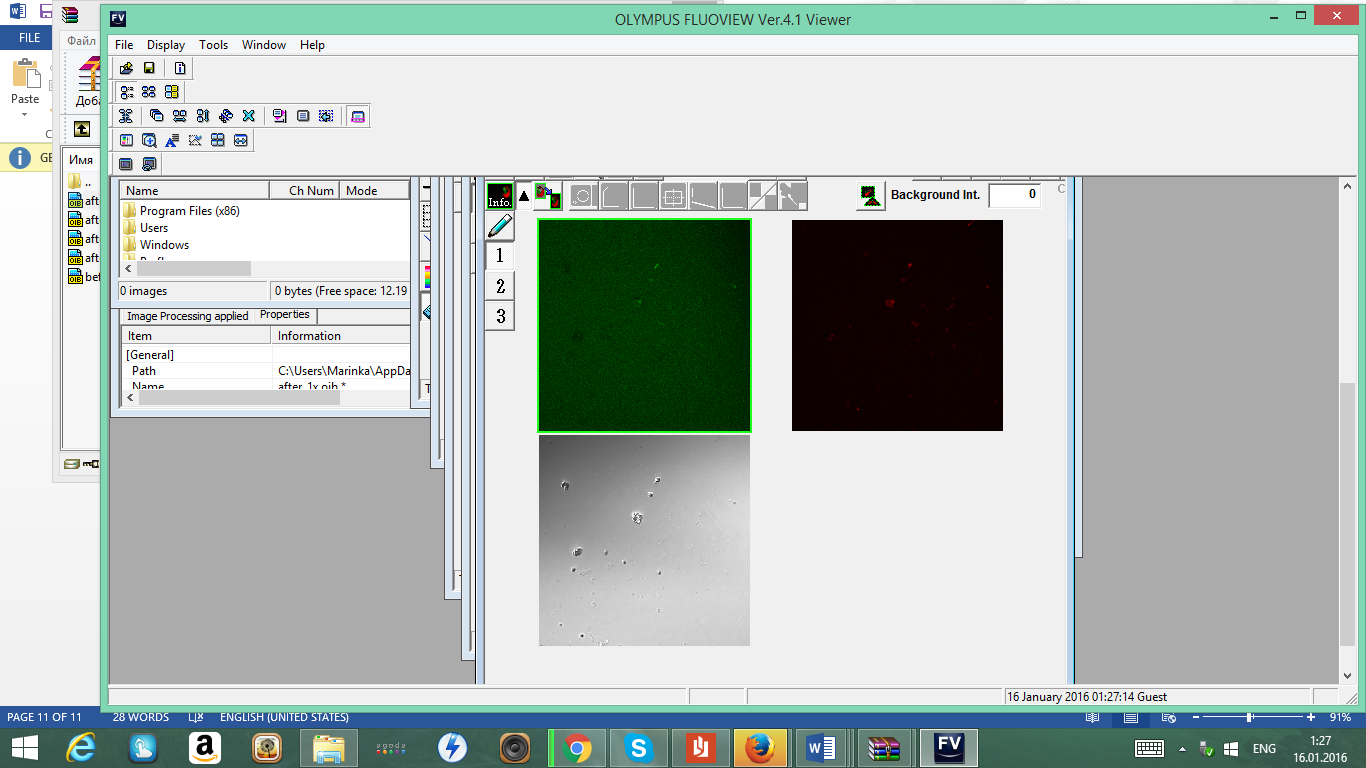


**(c)**

**Figure S5.** CSLM images of (a) (BSA-TA)_4_ microcapsules loaded with BSA-FITC by post-loading approach, immediately after their treatment (b) with 8M urea or (c) with simulated intestine fluid.

Amount of Lf incorporated in BSA-TA microcapsules have been analyzed by western blot. First, microcapsules were re-suspended in 8M Urea, 50mM Tris pH 8.9 and incubated for 1 h. Protein concentration was determined using the Bradford method [Bradford, M. M. A rapid and sensitive method for the quantitation of microgram quantities of protein utilizing the principle of protein-dye binding. Anal. Biochem. 72, 248-254 (1976)] and 1 μg, 0.4 μg, 0.2 μg and 0.1 μg of protein was separated on 10% BisTris NUPAGE gels (Life Technologies) with a dilution series of purified Lf (2.5 μg to 0.025 μg). The protein was transferred onto nitrocellulose membranes (Pall Corporation, East Hills, NY, USA). Membranes were blocked in Tris-buffered saline (TBS) solution (0.05 M Tris-HCl, 0.15 M NaCl, pH 7.6) containing 0.1% Tween 20 (TBST) and 5% BSA for 2 h. After three washes in TBST containing 0.1% BSA, membranes were incubated for 2 h in TBST containing 0.1% BSA with 1:60000 dilution of anti-Lf antibody (JWT88b, rabbit polyclonal antibody raised against purified bovine Lf). Following three further washes in TBST containing 0.1% BSA, membranes were incubated for 1 h in TBST containing 0.1% BSA with a 1:10000 dilution of goat anti rabbit secondary antibody conjugated to horseradish peroxidase (Sigma-Aldrich Co Ltd, Gillingham, United Kingdom). Finally membranes were washed in TBST containing 0.1% BSA three times and then washed a further four times in TBS. To visualize the immunoreactive bands, membranes were incubated for 1 min in ECL Western blotting detection reagents (Amersham, GE Healthcare, Buckinghamshire, United Kingdom) and then detected using the ImageQuant LAS 4000 imaging system (GE Healthcare Biosciences, Pittsburgh, PA, USA). The densities of immunoreactive bands were determined using Quantity One software (BioRad, Hercules, CA, USA).


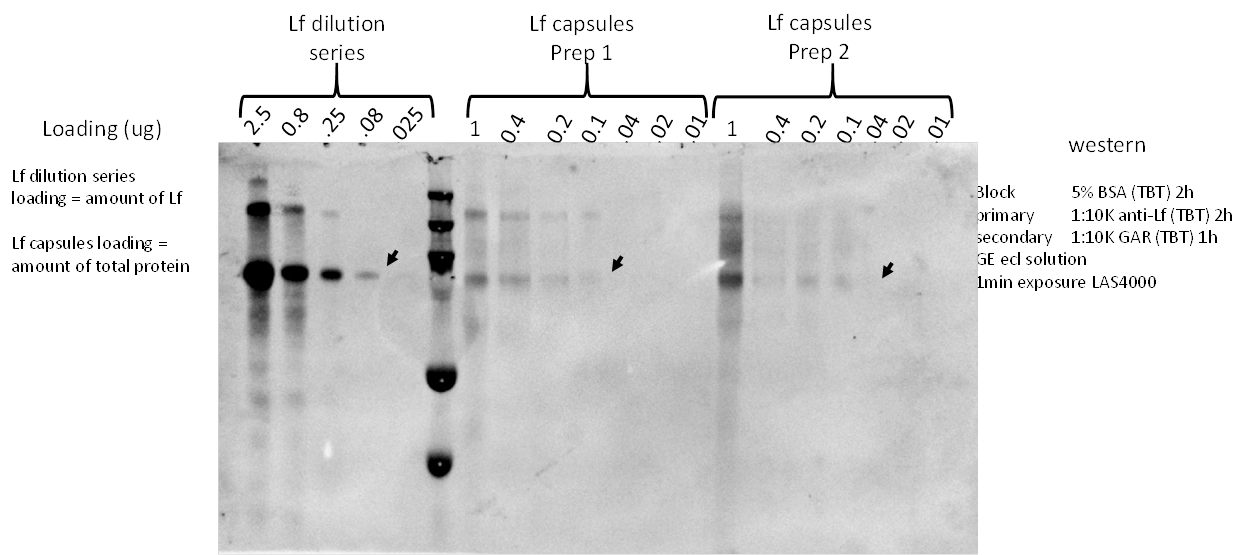


**Figure S6.** Western blot of standard Lf solutions (left – Lf dilution series) and a solution obtained after degradation of Lf/(BSA-TA)_4_ microcapsules with 8M urea (right – Lf in capsules Prep 1). Released amount of Lf was measured by densitometry was found to be 0.72 mg and total protein of 6 mg.

Time-of-flight secondary ions mass spectroscopy (ToF-SIMS) analysis was conducted using ToF-SIMS-IV instrument (ION-TOF GmbH, Germany). Positive secondary ions were detected, since Fe^+^ has a high yield of positive ions. For the measurement, a suspension of microcapsules was deposited on a copper substrate, dried in hot air (50-60 ^o^C) and put under vacuum. The copper was used here because the ion yield from a pure Cu surface is very low. To remove any surface contamination from air and evaporated solution, a second beam of 1 keV Ar^+^ with 12 nA current scanned over a 200 μm x 200 μm area was used. Disappearance of the organic secondary ions from the Cu surface upon sputtering, was chosen as a criterion for complete removal of the surface contamination. At the same time, such sputtering also removed the shell of the microcapsules and allowed analysis of their internal content. Then the analysis beam of 25 keV Bi^+^ with 1 pA average current was rastered over a 50 μm x 50 μm area and a mass spectrum was obtained from each pixel of this scan. Focusing of the analysis beam provided a lateral resolution better than 1 μm. The peaks corresponding to C and Fe positive ions were identified in the mass spectra, their intensities were integrated and plotted versus primary ion beam position. Thus, mass resolved images were obtained.


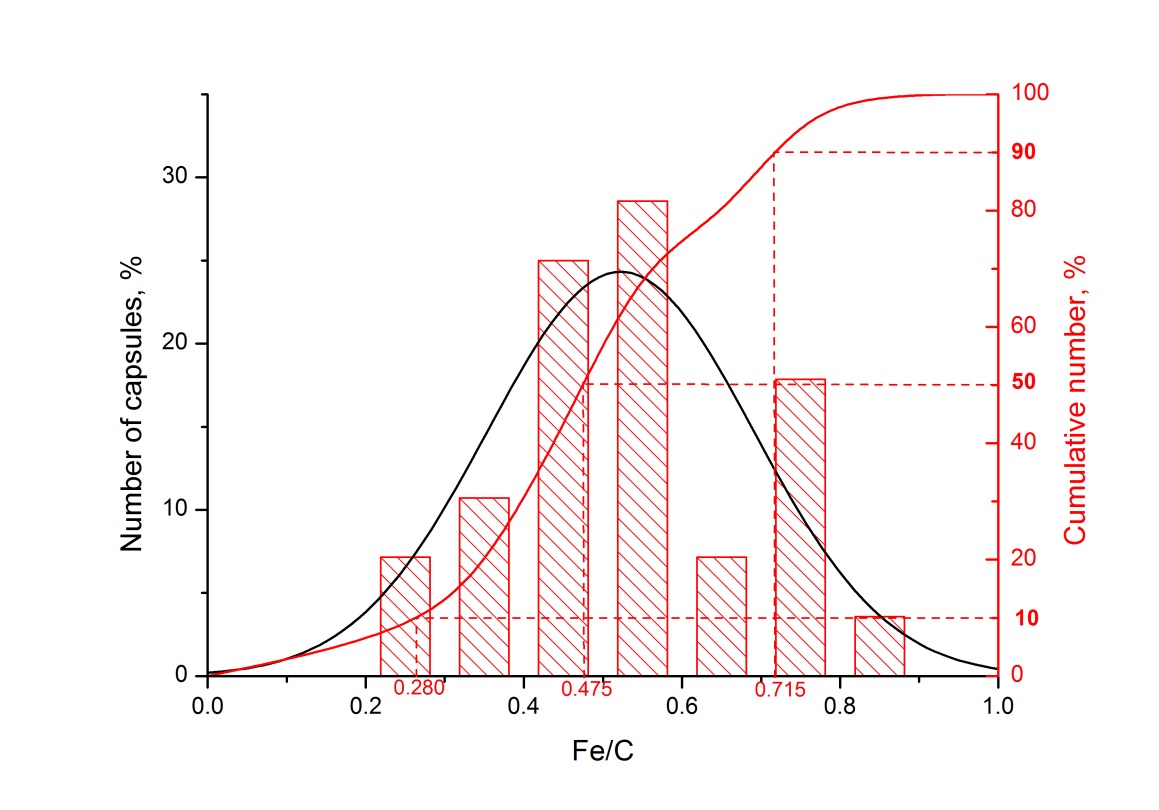


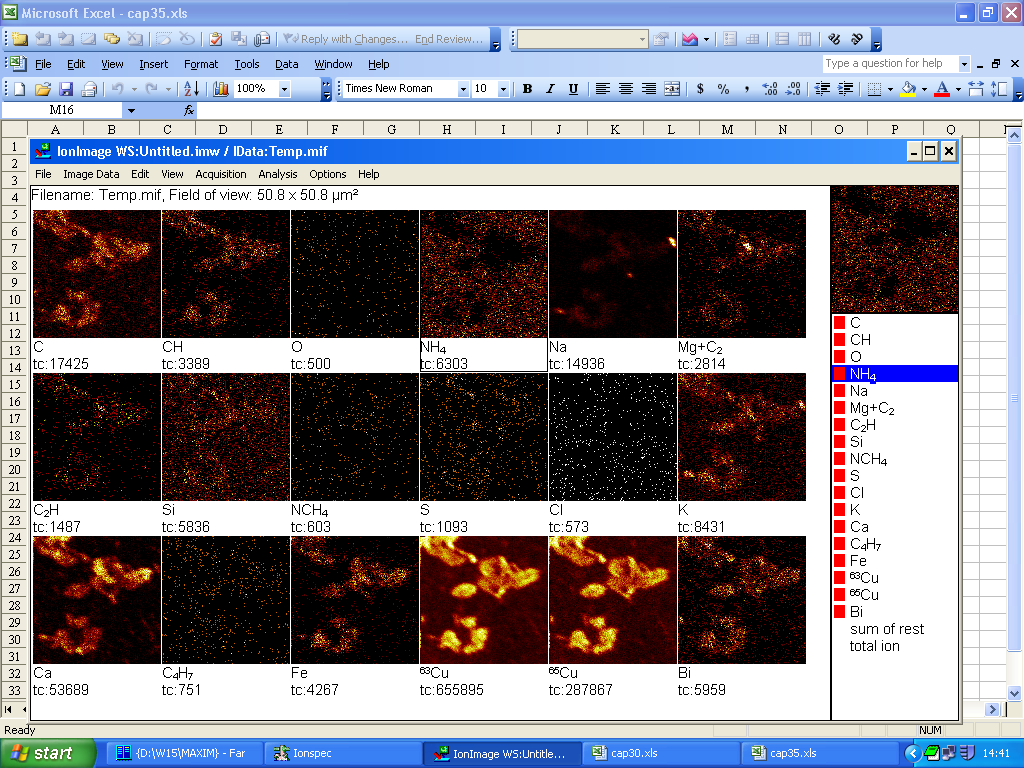

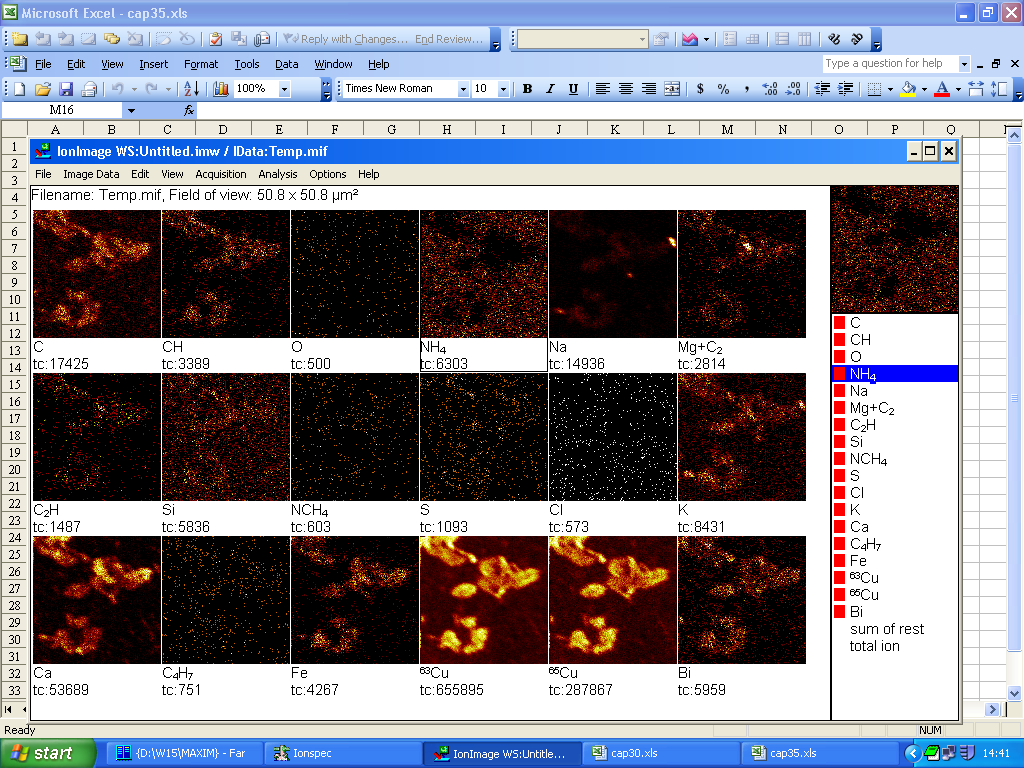


**a b**

**Figure S7**. (a) Mass resolved images (chemical maps) of positive ions from Lf-loaded microcapsules, scan size 50 μm x 50 μm; (b) histogram of Fe/C ratio showing distribution of Lf among individual microcapsules.


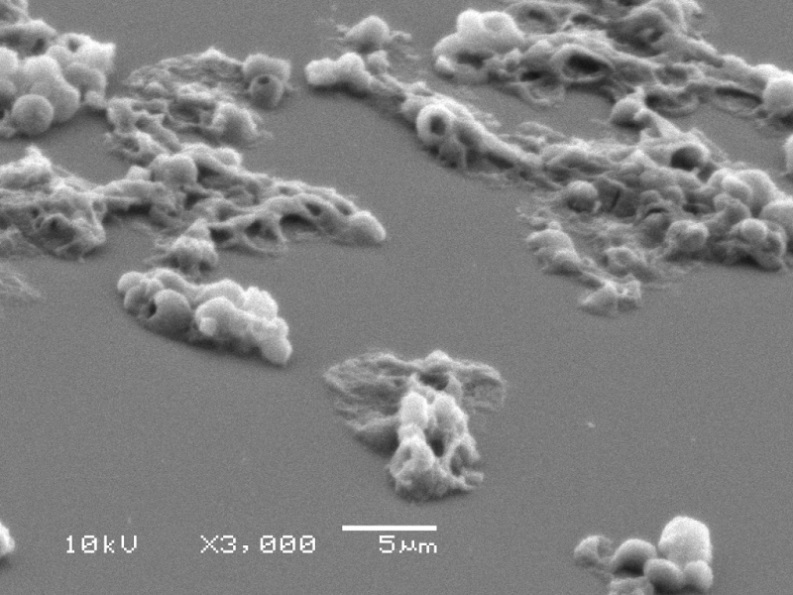


**Figure S8.** SEM images of the BSA-TA microcapsules with no Lf after CaCO_3_ cores dissolution. The microcapsules are partially collapsed.
